# Patients With Chronic Pain and Their Perspectives on Barriers and Opportunities for Earlier Opioid Tapering—A Qualitative Study Using Patient Journey Mapping

**DOI:** 10.1002/msc.70240

**Published:** 2026-06-18

**Authors:** Mette Dalsgård Bergmann, Stine Aalkjær Clausen, Anne Mette Schmidt, Peter Vedsted, Morten Hoegh, Malene Kjær Bruun, Heidi Guldberg Blæsbjerg, Camilla Blach Rossen

**Affiliations:** ^1^ Multidisciplinary Pain Centre, Elective Surgery Centre Silkeborg Regional Hospital Silkeborg Denmark; ^2^ Medical Diagnostic Centre University Clinic for Innovative Patient Pathways, Regional Hospital Central Jutland Silkeborg Denmark; ^3^ The Danish MS Hospitals Ry and Haslev Denmark; ^4^ Department of Clinical Medicine Aarhus University Aarhus Denmark; ^5^ Department of Health Science and Technology, Faculty of Medicine Aalborg University Aalborg Denmark; ^6^ Center for General Practice at Aalborg University Gistrup Denmark; ^7^ Regional Clinic Skjern Skive Denmark; ^8^ University Clinic for Interdisciplinary Orthopaedic Pathways, Elective Surgery Centre, Silkeborg Regional Hospital Silkeborg Denmark

**Keywords:** chronic pain, Denmark, drug tapering, opioid‐related disorders, qualitative research

## Abstract

**Purpose:**

Long‐term opioid use is associated at the population level with increased risks such as physical dependence, adverse effects and complex healthcare trajectories. While some patients report improved function and quality of life with carefully monitored opioid therapy, others experience diminishing benefits over time and substantial harms. While tapering is clinically recommended, little is known about patients' perspectives on barriers and opportunities for initiating earlier opioid tapering. This study explores how patients experience and reflect on these issues throughout their patient journey.

**Methods:**

We conducted semi‐structured interviews with 10 patients who had completed opioid tapering at a Danish multidisciplinary pain clinic. Patient journey mapping was used to visualise and structure individual treatment journeys. Data were analysed using reflexive thematic analysis and supported by NVivo software.

**Results:**

Two overarching themes emerged: (1) *living between fear of pain and dependence on opioids* included emotional ambivalence, fear of pain recurrence, and insufficient guidance from healthcare providers; (2) *Tapering responsibility falling through the cracks*, illustrated how tapering responsibility often became fragmented across the healthcare system, leaving participants feeling unsupported and uncertain about how to initiate change. Conversely, proactive engagement by healthcare professionals, particularly general practitioners, enabled participants to consider tapering.

**Conclusion:**

This study identified personal and systemic barriers to opioid tapering, including fear of pain, emotional dependence, and fragmented care. Participants favoured earlier tapering, but success depended on addressing these factors and enhancing general practitioners' knowledge. Meaningful tapering becomes possible when healthcare professional responsiveness meets individual readiness.

## 1 Introduction

The IASP's definition of chronic pain is pain lasting more than 3 months or beyond the expected healing time. Chronic pain is a multifaceted condition with physical, psychological and social impacts on the individual (International Association for the study of Pain 2020).

International guidelines explicitly advise against opioid use for chronic non‐cancer pain, and current clinical recommendations broadly emphasise minimising long‐term use and recommend tapering strategies when clinically appropriate and with individualised collaborative decision making (Danish Health Authority 2019; EFIC 2021; U.S. Centers for disease control and prevention, 2022; World Health Organisation 2023).

In Denmark, opioid use has increased for several years. Since 2016, targeted efforts, fuelled in part by growing awareness of the global opioid crisis, have led to a decline in overall use, including a reduction in the number of people in long‐term opioid use (more than six months) (Danish Health Data Authority 2023). Compared to countries like France and Norway, where opioid use for non‐cancer pain is more restricted, Denmark still shows relatively high prescription rates and was in 2019 ranked fifth in global opioid consumption (Danish Health Data Authority 2023, 2024; World Health Organisation 2023).

Limited access to alternative treatments, inconsistent information from healthcare providers, and fear of increased pain leave patients feeling trapped in long‐term opioid use (Alenezi et al. 2022). These barriers are further compounded by challenges within the healthcare system, where healthcare providers, particularly general practitioners (GPs), report feeling ill‐equipped to initiate opioid tapering, citing a lack of training and insufficient deprescribing guidelines (de Kleijn et al. 2024; Punwasi et al. 2022).

These challenges have increased attention to careful opioid use, highlighting the importance of initiating tapering early in the treatment process (Dunn et al. 2024; EFIC 2021). However, there are still gaps in the literature regarding patient‐identified missed opportunities for earlier tapering along their treatment trajectories (Frank et al. 2016; Young et al. 2025).

This study therefore explores patients with chronic pain and their perspectives on the barriers and opportunities for initiating earlier opioid tapering, with particular attention to when they experience missed opportunities for timely or more effective tapering.

## 2 Methods

### 2.1 Design

This qualitative study used semi‐structured interviews supported using patient journey mapping techniques to explore patients' experiences related to long‐term opioid use (Braun and Clarke 2022).

### 2.2 Setting and Participants

Participants were recruited from a multidisciplinary Pain Clinic in Denmark (Silkeborg Regional Hospital). The clinic receives approximately 300 patients annually, primarily referred from GPs (Sundhed.dk 2020). The inclusion criteria were patients who had used opioids for more than 6 months and had completed opioid tapering during treatment at the Pain Clinic. Several participants described previous unsuccessful tapering attempts, which were explored as part of their treatment histories. Participants were required to be 18 years or older and able to speak and understand Danish. Non‐resumption of opioid use was assessed by self‐report during the recruitment contact. We did not systematically track opioid use after the interview and therefore cannot exclude the possibility that some participants may have restarted opioid treatment later.

To reduce social desirability, participants were excluded if they were or had been a patient of the authors. Participants were also excluded if they had co‐morbidities preventing them from providing a detailed account of their patient journey.

Participants were recruited through convenience sampling (Ormrod 2019), which allowed us to recruit participants with relevant lived experiences to address our research questions. Data collection continued until the sample provided adequate richness and diversity to support the development of a reflexive thematic analysis. A total of 13 participants agreed to participate. This initial contact with participants was made either via a phone call or in person at the Pain Clinic, where a doctor or nurse informed the patients about the study. This approach aimed to create a sense of safety (Wadmann 2013). A follow‐up phone call by SAC, MDB, or HGB ensured that the participant understood the purpose of the study and met the inclusion criteria. If the participants agreed, an interview was scheduled at the time and location of their choice. Two participants withdrew; one never responded to the phone calls, and another because of lack of time. In total, 11 participants, ranging from 25 to 75 years with diverse chronic pain conditions, were interviewed. The first interview served as a pilot and was excluded from the final analysis, resulting in a final sample of 10 participants. Participants were offered the option to involve a relative, who supplemented responses when participants had difficulty recalling specific details. Participants were informed in advance that the interviews would last approximately 60 min. The interviews were conducted between February 1 and March 31, 2024.

### 2.3 Data Collection

#### 2.3.1 Pilot Interview

A semi‐structured interview guide was developed. The interview guide followed a funnel‐shaped structure, moving from broad questions about living with chronic pain to more specific reflections on opioid use and tapering. The first interview served as a pilot, with HGB as the primary interviewer and MDB and SAC as observers. The observers documented potential revisions to the interview guide and provided follow‐up questions at the end. Based on insights from the pilot, the interview guide was revised (Supporting Information S1: Appendix 1). The pilot interview was not included in the data material and therefore does not exist in Table 1.

**TABLE 1:** Overview of participant characteristics.

Informant	Gender	Age	Start of opioid	Indication	Initial opioid prescribed by	Tapering initiation
P 1	W	75	2007	Back pain	Surgeon	2023
P 2	M	25	2020	Trauma	Surgeon	2022
P 3	W	43	2021	Shoulder pain	General practitioner	2023
P 4	M	49	2016	Back pain	Private clinic	2021
P 5	M	62	2016	Back pain	Surgeon	2019
P 6	W	57	2014	Back pain	Pain clinic	2024
P 7	M	58	1990	Hand pain	General practitioner	2023
P 8	W	66	1978	Back pain	General practitioner	2022
P 9	W	57	1999	Back pain	General practitioner	2023
P 10	M	75	2008	Back pain	General practitioner	2022

#### 2.3.2 Interviews

Participants were encouraged to reflect on their treatment journey before the interview and instructed to make notes of key events, interactions, and treatment journeys. Interviews were conducted by three authors (SAC, MDB, or HGB) in pairs, with one author conducting the interview and the other acting as an observer. The observer ensured that all topics in the interview guide were addressed and, when relevant, added clarifying or follow‐up questions at the end of the interview. All interviews were audio‐recorded and transcribed.

#### 2.3.3 Patient Journey Mapping

We applied ‘patient journey mapping’ as a method to explore and visualise key phases, touchpoints, barriers and opportunities in the participant's journey (Davies et al. 2023). This approach enabled the integration of both retrospective and in‐the‐moment reflections (Davies et al. 2023).

**FIGURE 1:**
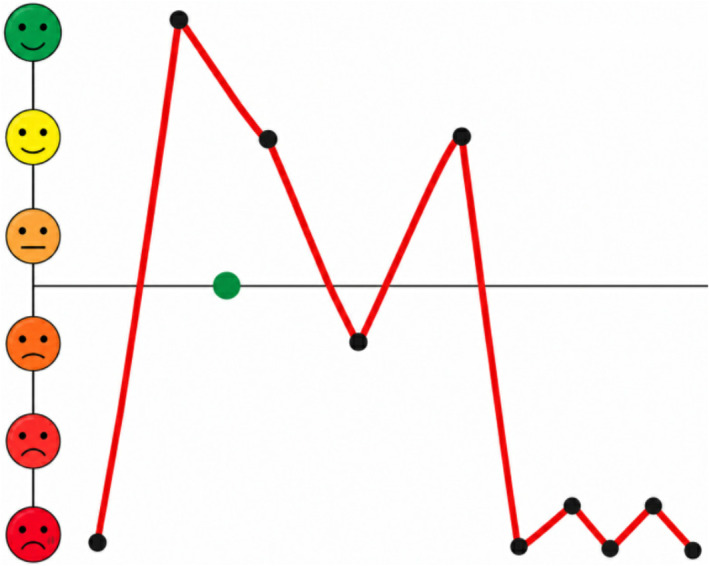
Composite illustration of a modified lifeline created by combining and adapting 10 individual lifelines to depict a general pattern. The horizontal axis represents time and the vertical axis indicates pain intensity (low to high; colour‐coded icons). Black nodes connected by the red line show the trajectory over time, and the green marker indicates the participant's retrospectively preferred timing of tapering.

As part of this process, a lifeline (Hunskår 2014) was introduced at the beginning of the interview. A printed A3‐sized version of the lifeline was placed on the table, allowing both the participant and interviewer to engage actively with it. The lifeline served as a visual aid to help participants organise their experiences and recall key events. Participants used the lifeline when describing essential moments such as the initiation of opioid use, treatment milestones, or significant encounters with healthcare providers, particularly focussing on the period from opioid initiation to the beginning of tapering ( Figure 1.

### 2.4 Data Analysis

Analysis was undertaken using reflexive thematic analysis (RTA) guided using Braun and Clarke's approach (2022). Transcribed data were analysed using NVivo software. In line with reflexive thematic analysis, we acknowledge that the analysis was shaped by the research team's professional backgrounds as physicians, physiotherapists, and registered nurses, their clinical experience with pain management and opioid treatment, and their interpretive engagement with the data. The authors have extensive experience treating patients with acute and chronic pain and have worked clinically with patients using opioids. One author is a nurse at a pain clinic with 8 years of experience supporting opioid tapering. The research team also included authors with extensive experience in qualitative research and reflexive thematic analysis. These backgrounds provided valuable contextual insight but also shaped the assumptions brought to the analysis. Through ongoing discussions among the authors, these assumptions were critically examined and alternative interpretations were actively explored throughout theme development. The analysis was therefore understood as an interpretive and reflexive process rather than as the discovery of objectively emerging themes.

In the familiarisation phase, SAC, MDB and HGB independently read and re‐read the interview transcripts and the lifelines to immerse themselves in the data and note preliminary impressions. During coding, MDB and HGB conducted exploratory coding of two interviews, identifying early codes such as ambivalence, timing, frustration and worries. As the analysis progressed, codes were revised, merged and restructured to capture the complexity of participants' narratives while retaining contextual nuance.

The full dataset was subsequently coded iteratively. The ongoing dialogue between the authors (SAC, MDB, AMS, PV and CBR) allowed for the refinement of codes and the development of broader meaning patterns. Patient journey mapping data (including participants' lifeline) were integrated into the analysis to add a temporal and visual dimension, enriching the interpretation of how tapering unfolds across time and healthcare interactions. This provided insight into timing and windows of opportunity for tapering.

Regular collaborative meetings enabled all authors to reflect on emerging patterns, challenge assumptions, and explore alternative interpretations. This iterative and dialogic process supported the development of coherent and distinctive themes that captured both diversity and commonality of the participants' experiences (Braun and Clarke 2022).

Next, emerging themes such as contacts, help/knowledge/communication/earlier opportunity, and effect/side effect/pain were refined through collaborative, interpretative discussions between authors. SAC, MDB, AMS, PV and CBR used a whiteboard to map and explore coherence, relationships, and boundaries between themes.

In the final interpretative phase, we returned to the dataset to ensure that each theme was grounded in the data. This iterative process of moving back‐and‐forth between data, codes and themes ensured that each theme represented a meaningful pattern of shared experiences and interpretation.

## 3 Results

**FIGURE 2:**
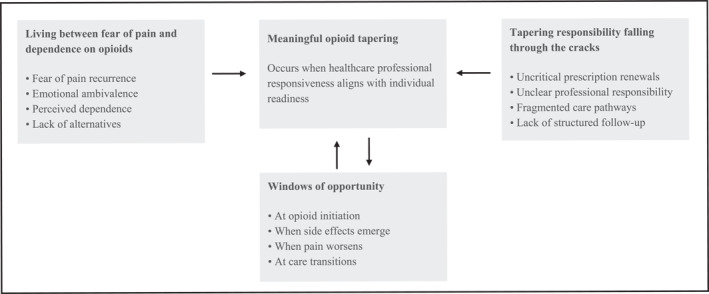
Conceptual model illustrating how meaningful opioid tapering becomes possible when healthcare professional responsiveness aligns with individual readiness. Tapering is shaped by the interaction between personal barriers, system‐level fragmentation, and windows of opportunity across the patient journey.

Through RTA, we developed two interpretive themes that captured how participants made sense of prolonged opioid use and the difficulties surrounding opioid tapering, (Figure 2). The first theme explored how fear of pain recurrence, perceived dependence on opioids, and emotional ambivalence contributed to continued opioid use. The second theme illustrated how tapering responsibility often became fragmented across the healthcare system, leaving participants feeling unsupported and uncertain about how to initiate change. Together, the themes reflected how continued opioid use was shaped through an interplay between personal experiences, clinical encounters, and structural conditions within the healthcare system.

### 3.1 Theme 1: Living Between Fear of Pain and Dependence on Opioids

This theme captured the complex set of barriers participants encountered when considering or attempting to initiate opioid tapering. We identified two interrelated subthemes: (1) Opioids as both relief and threat and (2) Feeling unable to taper despite wanting change. Although analytically separated for clarity, elements of both were entangled in participants' accounts.

#### 3.1.1 Opioids as Both Relief and Threat

At the onset of opioid use, all the participants described experiencing a pain‐reducing effect. The opioid was initiated during periods where the pain was described as severe and unbearable. For this reason, the idea of tapering opioids provoked strong emotional reactions, primarily driven by a fear of returning to that prior state of pain. P8 expressed:

[Interviewer asks: Can you feel that you needed it [opioids]?] Yes, I could feel that. Otherwise, I couldn’t function.

[Interviewer asks: Did it help you with the pain?] Yes, it did. But it also meant that I needed more and more, in some way… I have to honestly admit that I was, of course, also scared about whether I could even bear being in my body if I started tapering down……But just going through those one and a half years, thinking about having to taper down, and being a bit scared of it while at the same time wanting to do it, you know? That’s actually not very pleasant.’

Even though the effect of opioids diminishes over time, all participants remained reluctant to reduce. The persistent presence of pain combined with the memory of earlier relief made tapering feel irrational or even threatening. For some, attempts to reduce opioids were experienced as intrusive. P4 explained:

And then that nurse says… I remember she holds it [Oxycontin] back. That's when I started to get angry. Because she shouldn't start saying that I couldn't have it, I remember that. I'll never forget it.

Although many participants retrospectively recognised that prolonged opioid use was not ideal, they also described feeling incapable of tapering at the time. Fear, dependency, and a lack of perceived alternatives left them feeling unprepared to make a change.

#### 3.1.2 Feeling Unable to Taper Despite Wanting Change

##### 3.1.2.1 Prescription Renewal Is (Too) Easy

Nearly all the participants described how they had relatively easy access to prescription renewal with their GP, often without the need for a personal consultation. This lack of critical evaluation was perceived by many as a significant barrier to tapering.

P3 reflected on how, in hindsight, her GP gave her too much autonomy:

My GP has been really sweet because she gave me what I wanted to relieve my unbearable pain. I was really satisfied with her because I got what I needed when I said I needed my prescriptions renewed, and I needed to take so much, so I got it. But I can see, in hindsight, that I was given too much freedom. You need to be kept on a short leash, really under control. The GP should almost count the pills to make sure you don’t end up taking more.

Initially, this participant perceived the GP's support as helpful because it respected her autonomy. However, looking back, she reframed this experience as lacking the structure and oversight she now believes was needed. This tension between a need for autonomy and an unmet need for supported tapering was a recurring theme in data.

Several of the participants felt unsupported in their tapering journey and pointed to specific instances on the lifeline in front of them where guidance was lacking. Many described their GP as not having the necessary knowledge to initiate or manage tapering. P8 shared her frustration with the lack of support from healthcare professionals:

If the tapering had been done properly, I believe I would not have minded. But the issue is that support is needed. Yes, support is essential, and one must be certain that what they are doing is the right approach.

[Interviewer asks: Do you have any thoughts on tapering yourself?] Yes, I do. But I was also in severe pain all the time. So, it was also a question of… how does one go about it? That is where my GP said it is too difficult for her. She neither had the time, capacity, nor the necessary experience with tapering at such doses.

These accounts suggest that participants not only needed access to tapering but also relied on their GPs to have the confidence, time, and expertise to support the process, needs that many felt were unmet.

##### 3.1.2.2 Tapering May Be Underprioritised

Several participants had undergone surgery as part of their pain trajectory. At hospital discharge, guidelines recommend providing a tapering schedule. However, three participants reported never receiving such guidance, and two others received tapering plans that they found too difficult to follow. P6 explained:

Of course, I was followed up by the surgeons until they said it was fine. But they don’t get involved with the medication. I just couldn’t figure it [the tapering plan] out. After a couple of weeks, I had to call my GP and tell him, I simply didn’t understand it.

For many, it remained unclear who was responsible for supporting tapering after surgery.

P3 unsuccessfully attempted to manage tapering herself after discharge, as she did not get a tapering plan from the hospital.

There was absolutely no guidance whatsoever. As a result, I attempted to taper on my own and likely approached it too aggressively by reducing my dose by 10 mg three times a day. I simply cut down drastically, which left me completely unable to function. Then, I tried a more gradual approach, but I realized it was too easy to take a little more when there was no external oversight or support.

Taken together, participants' accounts illustrated how tapering was not merely a question of clinical readiness but shaped by a complex interplay of emotional fear, systemic inertia, and fragmented responsibility. Through our reflexive analysis, we identified a pattern in which fear of pain recurrence, combined with uncritical prescription renewal and insufficient professional guidance, created a cycle of continued opioid use. Although participants later questioned the appropriateness of their medication use, at the time, they felt unsupported, unprepared, and unsure where to turn for help. This underscored how tapering required not only patient motivation but also a coordinated and knowledgeable system of support, which participants experienced as largely absent.

### 3.2 Theme 2: Tapering Responsibility Falling Through the Cracks

This theme captured how responsibility for opioid tapering often became fragmented across participants' healthcare trajectories, leaving important opportunities for change unaddressed. Through our reflexive analysis, we identified patterns in how moments of readiness for tapering emerged, while meaningful support and coordinated follow‐up from healthcare professionals were often experienced as inconsistent or absent. Together, the subthemes illustrated how participants frequently found themselves navigating tapering without clear guidance or shared responsibility.

#### 3.2.1 Moments Where Change Became Imaginable

The participants retrospectively identified three particularly significant events in their trajectories when opioid tapering might have been more feasible or acceptable. The first concern was the initial prescribing of opioids. Several participants reflected that this moment presented a window of opportunity to discuss the temporary nature of opioid use, develop a tapering plan, and take a more holistic view of their situation. P6 stated the question concerning advice to other patients and healthcare professionals:

Well, you need to be informed about your rights and that you can actually learn to live with pain. There are some things that can help you without needing to rely entirely on medication…. There should be more discussion about that. Follow‐up on things, you know? So that it doesn’t take so many years, right? Right from the beginning, there should be more explanation about how to get through such a process. You might need to be more proactive about contacting your doctor because he probably won't reach out to you himself. There’s simply a lack of information from the start.

A second opportunity for earlier tapering appeared when participants began experiencing side effects. Several described the onset of side effects as a second missed opportunity, a point at which intervention might have prevented prolonged use and avoidable harms. These moments of discomfort or doubt often prompted reflection. P4 explained:

My cognitive abilities were quite compromised. I was completely out of it. During 2019–2020, things really spiralled……. I had one night where neither my wife nor I slept because I could just feel that if I fell asleep, something would go wrong. That was probably when I first started thinking… I need to do something about this, right? I was completely out of it, not present at all… It just caused things like not being able to sleep… Some of the typical side effects of opioids were definitely present. I did feel a bit uplifted. That’s also why I could function in a way. But cognitively, I was completely shut down at that time. So, I was aware that this wasn’t working, and I had to get out of it. I was really determined to get out of it.

Here, the physical and psychological impact of opioid use acted as a wake‐up call, prompting internal motivation to taper.

A third opportunity appeared when contacting the GP because of increasing pain despite being medicated with opioids. For some, this situation was interpreted as a signal that the medication was no longer effective and that change was required. This is described by P6 as an opportunity to initiate earlier opioid tapering:

Then I started to experience more pain again, and I thought, it can’t be right that I’m taking so many pills, and it still hurts.

[Interviewer: What did you think when the doctors said you might need to get off it?] I thought, if that’s what it takes, then that’s what it takes.

The mismatch between treatment and outcome created moments of doubt, often experienced as a tipping point where tapering might have been both meaningful and acceptable. However, whether such moments translated into action depended strongly on the support available from healthcare professionals. In some accounts, the GP encounter represented a potential turning point, but without proactive follow‐up or collaborative planning, this opportunity risked being lost.

#### 3.2.2 Fragmented Responsibility From the Healthcare Professionals

Participants' narratives pointed to healthcare professionals as potential catalysts for initiating opioid tapering. Particularly, when their involvement was timely, knowledgeable, and relationally attuned. We identified moments where contact with a health professional offered the opportunity for change. Several participants emphasised that timing, the point in their trajectory when information or challenge was introduced, played a decisive role in whether tapering became a viable option. As P1 noted, the shift in her treatment path occurred when she was assigned a new GP who had a different perspective on the treatment of chronic pain:

Now, I got a new GP, a young GP, and she has learnt something completely different. She is my salvation. With the change of GP, things started to happen. I actually think she's really skilled because she is willing to follow up on me.

This participant attributed the change in motivation not only to the GP's clinical knowledge but also to her willingness to follow up, suggesting that relational continuity and responsiveness were essential elements of supportive tapering.

Participants also mentioned other healthcare professionals, such as physiotherapists and chiropractors, as being involved in conversations about pain and function. However, there was often ambiguity around their role in relation to medication, and the participants did not report any dialogue with other healthcare professionals, besides their GP, about the importance of opioid tapering. When asking P5 if his physiotherapist ever questioned his medication, he replied:

She [the physiotherapist] asked about the medication I was taking. We had a dialogue about it. But she never expressed an opinion on how much or how little I was taking. No. It's not something I remember as being important. My physiotherapist looked at the whole person. And she engaged with it. The dialogue, the understanding, and the respect. And the fact that we listen to each other.

This account illustrated how other healthcare professionals provided meaningful interpersonal support but typically did not address opioid use directly. Their role was perceived as relationally important though structurally limited in terms of initiating change in medication practice.

## 4 Discussion

Our analysis highlighted how individual factors, both among patients and healthcare professionals, alongside systemic factors shape the barriers and facilitators for tapering. In the following section, we discuss two key aspects: The need for proactive healthcare professional engagement and gaps in system‐level support and guideline implementation.

### 4.1 The Need for Proactive Professional Engagement

Patients in our study did not reject the idea of tapering but emphasised the need for structured ongoing support. Many described lacking both information and encouragement, which prevented them from considering tapering as a realistic and safe option. This resonates with previous research, which highlighted that patients expressed significant concern about tapering without relational and social support (Frank et al. 2016), while other studies identified fear of increased pain and poor communication as key barriers to tapering (Quinlan et al. 2021). Similarly, we found that fear of unmanaged pain and insufficient communication with healthcare professionals were key barriers, underscoring the importance of early, proactive, and reassuring support. Participants valued continuity, relational trust, and the sense that tapering is something done *with* them and not *to* them. This reflects the emphasis on trust, open communication, and shared decision‐making in successful tapering identified in other studies (Danish Health Authority 2023; Frank et al. 2016). It also aligns with findings highlighting that individualised, compassionate communication, especially when tapering was done gradual and collaborative, was associated with more positive outcomes (Matthias et al. 2017).

GPs emerged as pivotal for the participants. Our findings suggest how automatic prescription renewals, without critical review or follow‐up, had enabled continued opioid use. In contrast, major turning points were often linked to encountering a new GP, who was perceived as more proactive, guideline‐informed, and willing to reassess treatment. These observations align with findings from Punwasi, whose qualitative systematic review found that more experienced GPs were often embedded in habitual prescribing cultures, whereas younger GPs demonstrated greater engagement with current guidelines and tapering strategies (Punwasi et al. 2022). These differences highlight the role of professional culture and underscore the need for continuous education and reflective practice to promote earlier intervention and safer prescribing.

The importance of early clinical engagement is further supported by findings from Neprash, who demonstrated that patients randomly assigned to GPs with high opioid prescribing were more likely to develop long‐term opioid use. Their study showed that even a single decision to prescribe opioids could have long‐lasting consequences, reinforcing the need for proactive, guideline‐informed decisions from the very beginning of treatment (Neprash and Barnett 2019). This further emphasises that tapering success is not only shaped by the patient's motivation but also by the GP's early decisions and their willingness to engage in structured, preventative approaches.

### 4.2 Gaps in System‐Level Support and Guideline Implementation

Although tapering of long‐term opioid use is widely recommended (Danish Health Authority 2019; World Health Organisation 2023), our findings reveal a persistent gap between these recommendations and clinical practice. For instance, the Danish Health Authority's guidelines recommend that GPs reassess opioid use every 2–3 months (Danish Health Authority 2019); however, participants described inconsistent follow‐up, unclear responsibilities between sectors, and a lack of structured plans. While some were offered tapering plans, others were left without any guidance, making tapering dependent on individual professionals rather than embedded in structured strategies. Tapering was often postponed until patients reached a crisis point.

System‐level interventions demonstrate that this gap can be closed. Meisenberg showed that multi‐component strategies including prescriber education, data transparency, patient information and leadership engagement reduced prescribing without compromising patient satisfaction (Meisenberg et al. 2018). Similarly, Howard found that implementing evidence‐based prescribing during clinical transitions, such as postoperative care, significantly reduced opioid use (Howard et al. 2020). Our participants likewise identified surgical episodes and other care transitions as missed opportunities, underscoring the need for proactive, guideline‐based engagement by healthcare professionals in transitions.

Finally, broadening responsibility beyond GPs may strengthen tapering support. Wenger highlights physiotherapists' expertise in pain management, pacing, and education, positioning them as valuable contributors to interdisciplinary pain teams (Wenger et al. 2018). Integrating physiotherapists earlier may provide patients with timely, non‐pharmacological strategies that reduce the likelihood of long‐term use.

System‐level change with clear procedures, defined responsibilities and cross‐sectoral coordination is essential to ensure that tapering is not only recommended but realistically implemented.

### 4.3 Methodological Considerations

Our results should be considered in the light of several methodological considerations. Revisiting painful or complex experiences is inherently subjective (Malterud 2001; Osborn and Rodham 2010) and is shaped by factors such as memory, emotional states, and perceived saliency of events (Gendreau et al. 2003). To support participants in remembering and structuring their narratives and identifying key moments in their opioid trajectories, interviews incorporated a visual lifeline, complemented by funnel‐shaped questioning that gradually guided the interview from general experiences to specific reflections. Nevertheless, stigma related to long term opioid use and pain may have influenced what participants chose to share, a recognised concern according to the literature (Cheetham et al. 2022; Osborn and Rodham 2010).

All participants had undergone opioid tapering at the same specialised pain clinic, providing a shared experiential context while allowing for variation in symptom burden and duration of opioid use. This combination supported the development of rich nuanced accounts and strengthened the analytic depth by illuminating differences across individual trajectories (Ahmed 2024). In reflexive thematic analysis, the purpose is to generate an in‐depth interpretive understanding rather than statistical generalisability. Sample size is therefore guided by the richness and relevance of the data in relation to the study aim (Braun and Clarke 2022). The 10 participants in this study provided sufficiently rich and detailed accounts to support the development of meaningful themes. Because recruitment was conducted consecutively during routine clinical follow‐up, we did not systematically record the total number of eligible patients. We are therefore unable to determine how closely the participants reflect the broader population of patients undergoing opioid tapering. However, the aim of this qualitative study was to generate in‐depth interpretive insights rather than statistical representativeness.

In line with a reflexive thematic analysis approach, the findings are understood as situated and interpretative, developed through active engagement between researchers and data, rather than as objective or universally representative truths. The use of convenience sampling and a single clinical setting means that the findings are most applicable to similar specialist pain contexts. However, detailed contextual and analytic descriptions are provided to support readers in assessing the potential relevance of the findings to other settings, including primary care.

Our inclusion criteria captured patients who had not resumed opioids at the time of recruitment, but later resumption could not be assessed, which may limit our understanding of the long term sustainability of tapering.

The authors engaged in ongoing reflective discussions throughout the analytic process, which supported strengthening the study's dependability and rigour (Braun and Clarke 2022). One author was affiliated with the Pain Clinic where data collection took place. This insider perspective contributed contextual insight, while reflexive discussions and involvement of external researchers helped ensure that interpretations were critically examined and not limited to a single perspective.

## 5 Conclusion

Our study extends existing work by focussing specifically on how patients retrospectively identify barriers and earlier opportunities for tapering mapped across their treatment journeys.

Participants retrospectively expressed a wish for earlier tapering, but also described ambivalence, shaped by fear of pain recurrence, dependency, and lack of perceived alternatives. These personal factors were compounded by healthcare professionals and systemic issues, including uncritical prescription renewals, fragmented care pathways, and lack of coordinated tapering support.

These perspectives reflect that tapering plans could be beneficially introduced from the outset of opioid treatment. However, earlier tapering requires more than the participants readiness; it depends on a complex interplay of personal, relational, and systemic factors. Strengthening GP's knowledge and engagement with tapering is essential, while other healthcare professionals, such as physiotherapists, may play a more predominant role in supporting non‐pharmacological pain strategies.

Ultimately, meaningful tapering becomes possible when healthcare professional responsiveness aligns with individual readiness.

## Author Contributions

**Mette Dalsgård Bergmann:** conceptualization, methodology, data curation, formal analysis, investigation, project administration, resources, writing – original draft. **Stine Aalkjær Clausen:** conceptualization, methodology, data curation, formal analysis, investigation, project administration, writing – original draft. **Anne Mette Schmidt:** conceptualization, methodology, supervision, validation, writing – review and editing. **Peter Vedsted:** conceptualization, methodology, supervision, validation, writing – review and editing. **Morten Hoegh:** conceptualization, methodology, project administration, resources, supervision, validation, visualization, writing – review and editing. **Malene Kjær Bruun:** conceptualization, methodology, data curation, validation, writing – review and editing. **Heidi Guldberg Blæsbjerg:** conceptualization, methodology, data curation, formal analysis, investigation, project administration, writing – review and editing. **Camilla Blach Rossen:** methodology, formal analysis, supervision, writing – original draft, writing – review and editing.

## Ethics Statement

According to Danish regulations, the study did not fall within the scope of the Danish Medical Research Involving Human Subject Act (§14) (Committees, 2025). The study adhered to the ethical principles outlined in the Helsinki Declaration (Association, 2013). All participants provided informed consent and were assured of confidentiality and the voluntary nature of their participation. All data were stored and handled in complete confidentiality.

## Conflicts of Interest

The authors declare no conflicts of interest.

## Supporting information


Supporting Information S1

## Data Availability

The data that support the findings of this study are available on request from the corresponding author. The data are not publicly available due to privacy or ethical restrictions.
