# Supplementary material for: Patients With Chronic Pain and Their Perspectives on Barriers and Opportunities for Earlier Opioid Tapering—A Qualitative Study Using Patient Journey Mapping
Source: Musculoskeletal Care. 2026 Jun 18;24(2):e70240. doi: 10.1002/msc.70240 (PMC13279846; doi:10.1002/msc.70240)
Supplement: Supplementary file 1 — Supporting Information S1 [file MSC-24-e70240-s001.pdf]

Appendix 1. Interview Guide

Theme	Main Questions	Probing / Follow-up Questions
Introduction	Introduce the life line to the interview participant.	During the interview, we will place your treatment course on this life line. The horizontal line represents the time from when you started taking opioids until you were referred for tapering at the Pain Clinic in Silkeborg, and the vertical line represents the development of your pain.
Presentation of the Informant	Would you like to start by telling me a little about yourself?	Name<br>Age<br>Family<br>Occupation
Patient Background	How long have you had pain?	
Start of Opioid Treatment	Why did you start taking opioids?	Do you remember when that was?<br>Who initiated the treatment?<br>Pain level + life line: How was your pain level in the period immediately afterwards?
Healthcare Contacts	Which healthcare professionals have you been in contact with throughout your entire course of treatment?	When did you have these contacts?<br>What was your experience with these healthcare contacts?
Healthcare Contacts	Was there any healthcare professional who talked to you about your morphine use?	For example: physician, nurse, physiotherapist, chiropractor, psychologist, psychiatrist, pain clinic, or others<br>Pain level + life line
Effect of Morphine	How did you experience the effect of opioids?	Side effects? Positive effects?<br>When did you notice these effects?<br>Pain level + life line
Opioid Tapering	Why did you start tapering off opioids?	Have you previously considered tapering or

		stopping?<br>Do you think you should have received help earlier?<br>Pain level + life line
Overall Reflections	What is your overall impression of your course of treatment?	What would you change?<br>What might others in a similar situation need? Why?
Closing	Is there anything else you would like to say or ask before we end the interview?	
